# Gut Microbiota-Dependent Trimethylamine N-Oxide Associates With Inflammation in Common Variable Immunodeficiency

**DOI:** 10.3389/fimmu.2020.574500

**Published:** 2020-09-16

**Authors:** Magnhild E. Macpherson, Johannes R. Hov, Thor Ueland, Tuva B. Dahl, Martin Kummen, Kari Otterdal, Kristian Holm, Rolf K. Berge, Tom E. Mollnes, Marius Trøseid, Bente Halvorsen, Pål Aukrust, Børre Fevang, Silje F. Jørgensen

**Affiliations:** ^1^Division of Surgery, Inflammatory Diseases and Transplantation, Research Institute of Internal Medicine, Oslo University Hospital, Rikshospitalet, Oslo, Norway; ^2^Section of Clinical Immunology and Infectious Diseases, Department of Rheumatology, Dermatology and Infectious Diseases, Oslo University Hospital, Rikshospitalet, Oslo, Norway; ^3^Faculty of Medicine, Institute of Clinical Medicine, University of Oslo, Oslo, Norway; ^4^Department of Transplantation Medicine, Norwegian Primary Sclerosing Cholangitis (PSC) Research Center, Oslo University Hospital Rikshospitalet, Oslo, Norway; ^5^Section of Gastroenterology, Department of Transplantation Medicine, Oslo University Hospital Rikshospitalet, Oslo, Norway; ^6^Faculty of Health Sciences and K.G. Jebsen Thrombosis Research and Expertise Center (TREC), University of Tromsø, Tromsø, Norway; ^7^Department of Microbiology, Oslo University Hospital HF, Rikshospitalet, Oslo, Norway; ^8^Department of Oncology, Oslo University Hospital HF, Oslo, Norway; ^9^Department of Clinical Science, University of Bergen, Bergen, Norway; ^10^Department of Immunology, Oslo University Hospital, University of Oslo, Oslo, Norway; ^11^Research Laboratory, Nordland Hospital, Bodø, Norway; ^12^Centre of Molecular Inflammation Research, Norwegian University of Science and Technology, Trondheim, Norway

**Keywords:** TMAO, CVID, gut microbiota, immunodeficiency, inflammation, *CutC*, *CntA*, diet

## Abstract

A substantial proportion of patients with common variable immunodeficiency (CVID) have inflammatory and autoimmune complications of unknown etiology. We have previously shown that systemic inflammation in CVID correlates with their gut microbial dysbiosis. The gut microbiota dependent metabolite trimethylamine N-oxide (TMAO) has been linked to several metabolic and inflammatory disorders, but has hitherto not been investigated in relation to CVID. We hypothesized that TMAO is involved in systemic inflammation in CVID. To explore this, we measured plasma concentrations of TMAO, inflammatory markers, and lipopolysaccharide (LPS) in 104 CVID patients and 30 controls. Gut microbiota profiles and the bacterial genes *CutC* and *CntA*, which encode enzymes that can convert dietary metabolites to trimethylamine in the colon, were examined in fecal samples from 40 CVID patients and 86 controls. Furthermore, a food frequency questionnaire and the effect of oral antibiotic rifaximin on plasma TMAO concentrations were explored in these 40 patients. We found CVID patients to have higher plasma concentrations of TMAO than controls (TMAO 5.0 [2.9–8.6] vs. 3.2 [2.2–6.3], *p* = 0.022, median with IQR). The TMAO concentration correlated positively with tumor necrosis factor (*p* = 0.008, rho = 0.26), interleukin-12 (*p* = 0.012, rho = 0.25) and LPS (*p* = 0.034, rho = 0.21). Dietary intake of meat (*p* = 0.678), fish (*p* = 0.715), egg (*p* = 0.138), dairy products (*p* = 0.284), and fiber (*p* = 0.767) did not significantly impact on the TMAO concentrations in plasma, nor did a 2-week course of the oral antibiotic rifaximin (*p* = 0.975). However, plasma TMAO concentrations correlated positively with gut microbial abundance of *Gammaproteobacteria* (*p* = 0.021, rho = 0.36). Bacterial gene *CntA* was present in significantly more CVID samples (75%) than controls (53%), *p* = 0.020, potentially related to the increased abundance of *Gammaproteobacteria* in these samples. The current study demonstrates that elevated TMAO concentrations are associated with systemic inflammation and increased gut microbial abundance of *Gammaproteobacteria* in CVID patients, suggesting that TMAO could be a link between gut microbial dysbiosis and systemic inflammation. Gut microbiota composition could thus be a potential therapeutic target to reduce systemic inflammation in CVID.

## Introduction

Common variable immunodeficiency (CVID) is the most common symptomatic primary immunodeficiency among adults with an estimated prevalence of between 1:25,000 and 1:50,000 (1), comprising a clinically and immunologically heterogeneous group. A maturation defect in the B-cell development to plasma cells results in impaired production of immunoglobulins (Ig), leading to the clinical hallmark of CVID; recurrent infections with encapsulated bacteria in the respiratory tract. In addition, ~70% of CVID patients have autoimmune and inflammatory complications (2, 3), associated with persistent systemic inflammation and immune activation, reflecting abnormalities in other immune cells such as monocytes/macrophages and T-cells (4). At present, however, the mechanisms leading to systemic sterile inflammation and autoimmunity in CVID are not fully understood.

The gut microbiota composition has been linked to systemic inflammation and metabolic disturbances in various systemic metabolic and autoimmune disorders (5–7). We have previously shown that gut microbial composition is related to systemic inflammation and autoimmunity in CVID. Particularly, the relative abundance of certain key groups of bacteria, which included increased levels of *Gammaproteobacteria*, were associated with systemic immune activation in CVID (8). However, the mechanisms by which an altered gut microbiota may translate into systemic inflammation in these patients are not clear.

**Figure 1:**
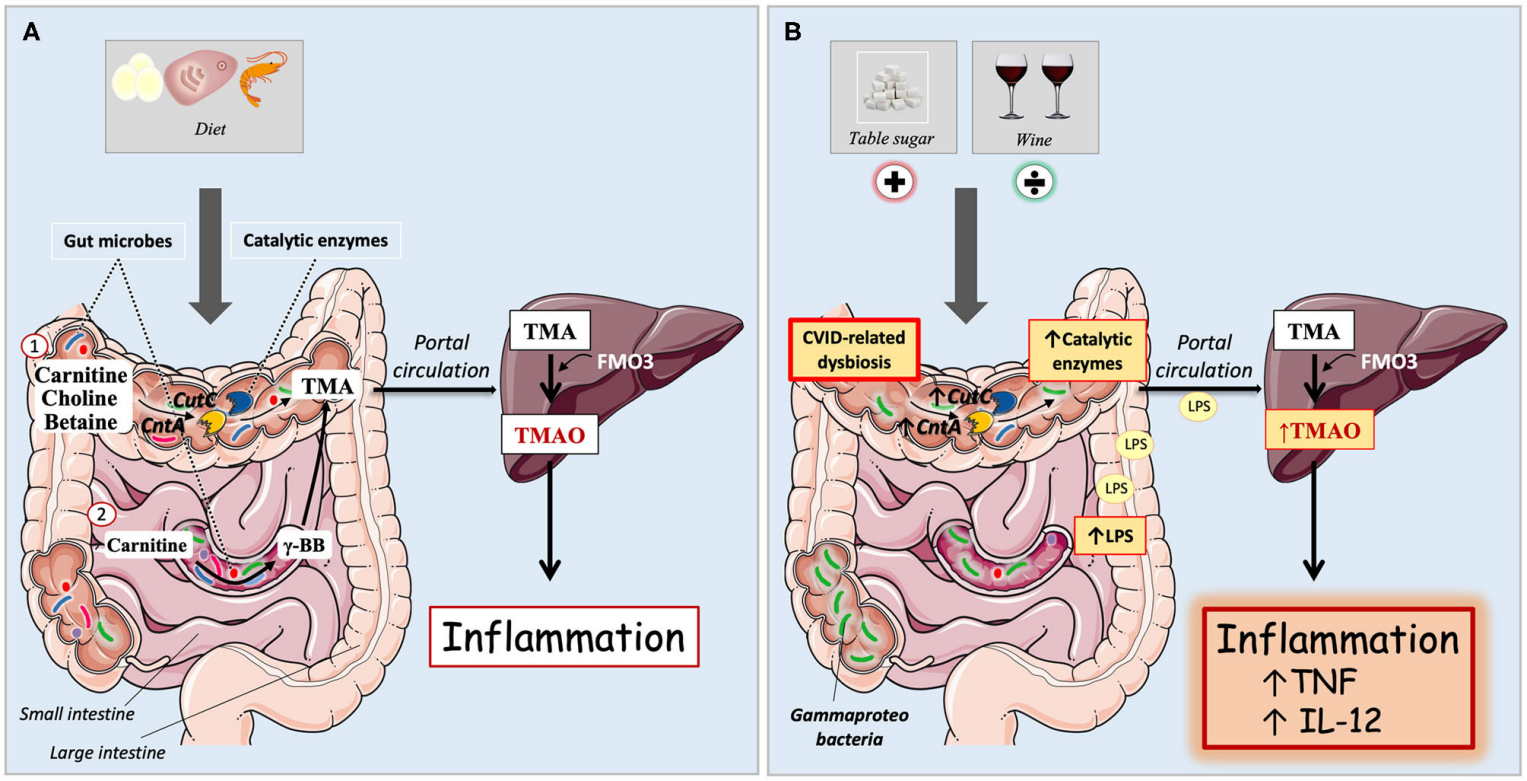
**(A,B)** Microbiota dependent pathways for trimethylamine N-oxide (TMAO) formation in immunocompetent individuals and CVID patients. **(A)** Immunocompetent individuals. Carnitine, betaine, and choline are mostly acquired through diet, and are metabolized by gut microbes to the TMAO precursor trimethylamine (TMA). Diet containing TMAO precursors are here symbolized by egg, meat, and shellfish. (1) Bacterial enzymes can convert dietary metabolites to TMA in the colon. These catalytic enzymes are encoded by bacterial genes, e.g., *CutC* (choline-TMA lyase) and *CntA* (carnitine oxygenase). (2) Carnitine can also be converted to the intermediate metabolite γ-butyrobetaine (γBB) in the small intestine, before conversion to TMA in the colon. TMA is passively absorbed from the gut to the portal circulation and delivered to the liver, where it is oxidized by flavin-containing monooxygenases (predominantly FMO3) to TMAO. Increased plasma levels of TMAO has previously been linked to systemic inflammation. **(B)** In CVID, there is an abundance of *Gammaproteobacteria* in the gut as part of an overall gut microbial dysbiosis. They have increased levels of catalytic enzymes *CutC* and *CntA* in the gut and elevated concentrations of TMAO in plasma. Increased levels of LPS correlate with raised TMAO, further associating with increased inflammatory markers TNF and IL-12. Dietary intake of table sugar correlates positively with TMAO whereas intake of red wine has a negative association with TMAO.

A number of studies, primarily in atherosclerosis and related metabolic disorders, have shown an association between the gut microbiota dependent trimethylamine N-oxide (TMAO) and systemic inflammation (9–11). The TMAO precursor trimethylamine (TMA) can be formed from dietary metabolites via microbiota dependent pathways in the gut. Bacterial enzymes such as carnitine oxygenase (*CntA*) and choline-TMA lyase (*CutC*) can convert carnitine and choline to TMA in the colon (12). Carnitine can also be converted to the intermediate metabolite γ-butyrobetaine (γBB) in the small intestine, before conversion to TMA in the colon (Figure 1A) (13). Meat, fish, and eggs are particularly rich in choline, whereas red meat and dairy products are the main sources of carnitine from the diet. In addition, carnitine can be endogenously synthesized from trimethyllysine via γBB (14). TMA is subsequently absorbed from the colon and translocated to the liver via the portal circulation, where it is oxidized to trimethylamine N-oxide (TMAO) by flavin-containing monooxygenases (Figure 1A).

We hypothesized that TMAO could represent a link between altered gut microbiota and systemic inflammation in CVID. To explore this hypothesis, we measured TMAO and other metabolites in the carnitine-TMAO pathway in CVID patients and healthy controls, and compared these metabolites to markers of systemic inflammation, gut microbiota composition and diet. In addition, we explored bacterial genes suggested to encode catalytic enzymes involved in the TMA production; *CutC* and *CntA*, the latter known to display high identities to *Gammaproteobacteria*.

## Materials and Methods

### Ethics

The Regional Committee for Medical and Research Ethics approved the study protocol. All study participants signed a written, informed consent. The work described has been carried out in accordance with the Declaration of Helsinki.

### Patient and Control Cohorts

CVID patients were recruited from the outpatient clinics at the Section of Clinical Immunology and Infectious Diseases, Oslo University Hospital, Rikshospitalet. CVID was defined as decreased serum levels of IgG, in addition to IgA and/or IgM by a minimum of two standard deviations below the mean for age, and exclusion of other causes of hypogammaglobulinemia (Primary immunodeficiency diseases. Report of an IUIS Scientific Committee. International Union of Immunological Societies. Clin Exp Immunol. 1999) (15). CVID subgroups were classified as “Infection only” or “Complications” based on criteria previously defined (3), however, CVID enteropathy was defined as persistent diarrhea after exclusion of gastrointestinal infection (8).

### Blood Sampling Protocol

Peripheral venous blood was collected into sterile blood collection tubes with EDTA as an anticoagulant. The tubes were immediately immersed in melting ice and subsequently centrifuged within 15 min at 2,000 g for 20 min to obtain platelet-poor plasma. Plasma was stored at −80°C in multiple aliquots.

### Analyses of Inflammatory Markers

Plasma levels of tumor necrosis factor (TNFα) and interleukin (IL)-6, IL-8, and IL-12 were analyzed using a multiplex cytokine assay (Bio-Plex Human Cytokine Plex Panel; Bio-Rad Laboratories Inc., Hercules, CA). The samples were analyzed on a Multiplex Analyzer (Bio-Rad Laboratories) according to instructions from the manufacturer.

### Measurement of Carnitine, TMAO, and Related Metabolites

Free carnitine and γBB were analyzed in plasma using MS/MS as described previously (16) with some modifications of the high-performance liquid chromatography conditions (Supplementary Methods). Stable isotope dilution liquid chromatography–tandem mass spectrometry (LC/MS/MS) was used for the quantification of TMAO, choline, and betaine in the same plasma samples (Supplementary Methods).

### LPS Analysis

LPS was analyzed by Limulus Amebocyte Lysate chromogenic assay (Lonza, Walkersville, MD) according to the manufacturer's instructions, with the following modifications: In the 104 CVID patients and 30 controls, samples were diluted 10-fold to avoid interference with background color, and preheated to 68°C for 10 min prior to analyses to dissolve immune complexes as previously described (8, 17).

### Microbiota Analyses

Participants collected stool samples at home within 24 h prior to their hospital visit, or alternatively at the hospital, with a standardized collection device (18). The stool samples were then transferred by the participants to stool collection tubes with Stool DNA Stabilizer (Stratec Biomedical, Birkenfeld, Germany) (19). Samples were stored at minimum −20°C according to the manufacturer's recommendations until DNA extraction. Bacterial DNA was extracted using the PSP Spin Stool DNA Plus Kit (Stratec) before being subjected to amplification of the 16S ribosomal RNA gene with dual-indexed barcodes according to an established protocol (20), followed by sequencing on an Illumina MiSeq (San Diego, CA; Supplementary Methods).

To assess bacteria with TMA producing potential we applied qPCR DNA assays targeting *CntA* and *CutC* using modified primers described by Rath et al. (12). Briefly, 30 ng DNA was analyzed using HOT FIREPol EvaGreen qPCR Mix Plus (Solis, BioDyne, Brumath Cedex, France) and Applied Biosystems 7900HT, primer concentration and thermal cycles as described (12). All products in the melting curve with a melting temperature above 78°C were considered a *CntA* product. *CutC* levels were normalized to 16S rRNA gene levels and presented as ddCt.

### Food Frequency Questionnaire

CVID patients were asked to complete a self-administrated, validated Norwegian food frequency questionnaire designed to reflect dietary habits over the past year (21, 22). The questionnaire offers multiple choice alternatives and also the opportunity to provide supplementary information regarding specific dietary restrictions or habits. It covers 180 food items and has serving size alternatives specified in households units, which is then converted to grams per day using a software developed at the Institute for Nutrition Research, University of Oslo (21).

### Statistical Analyses

The datasets for TMAO pathway metabolites were not normally distributed and so the data was log transformed. Where normal distribution was achieved, we continued with multivariate testing and where normal distribution was not achieved, we applied Mann–Whitney non-parametric testing. Fisher's exact test was used for categorical variables. Depending on the data distribution, we performed correlation analyses with either Spearman's rank correlation test (correlation coefficient rho) or Pearson's correlation test (correlation coefficient *r*). Additionally, stepwise linear regression analysis was performed to adjust for age and sex. For the longitudinal data, we compared datasets from three different time points using the Friedman test. Univariate Repeated measures ANOVA (UNIANOVA) was used to assess the effect of treatment (rifaximin), focusing on the interaction between time and treatment group for TMAO. Calculations were performed in SPSS (version 24, IBM, NY).

## Results

### Patient Characteristics

We consecutively collected plasma samples from 104 CVID patients over 13 months from the outpatient clinic at Section of Clinical Immunology and Infectious Diseases, Oslo University Hospital Rikshospitalet, and included 30 healthy controls as previously described (8). In addition, stool samples, blood samples, and food frequency questionnaire (FFQ) from 40 CVID patients (FFQ = 38 patients) from a preceding intervention trial (23) were included for sub-analysis. In CVID patients included for gut microbiota analyses, the use of antibiotics in the last 12 weeks was an exclusion criterion. Patients' characteristics are given for both cohorts in Table 1, Supplementary Table 1, and Supplementary Methods. Thirty-two CVID patients were included in both cohorts, but at different time points (8, 23). The 86 controls used in the analyses of *CutC* and *CntA* in stool samples did not have corresponding blood samples (Supplementary Methods).

**Table 1:** Background characteristics for CVID cohorts and healthy controls.

	**Main cohort**			**Subset cohort^d^**
	**CVID**	**Controls**	***P*-value^b^**	**CVID**
	***n* = 104**	***n* = 30**		***n* = 40**
Age in years, mean± SD	46 ± 15	47 ± 13	0.73^c^	48 ± 12
Female, *n* (%)	51 (49)	14 (47)	0.84^d^	25 (63)
BMI, mean ± SD	24 ± 4	24 ± 3	0.97^c^	26 ± 5
IVIG^a^, *n* (%)	19 (18)	–	–	6 (15)
SCIG, *n* (%)	76 (73)	–	–	30 (75)
IVIG and SCIG, *n* (%)	7 (7)	–	–	4 (10)
Infection only, *n* (%)	25 (24)	–	–	8 (20)
Non-infectious complications, *n* (%)	79 (76)	–	–	32 (80)

*IVIG, Intravenous immunoglobulins; SCIG, Subcutaneous immunoglobulin; BMI, Body Mass Index*.

a *Two CVID patients in the Main cohort did not receive any immunoglobulin substitution*.

b *P-value is given for CVID (n = 104) vs. Controls*.

c *Mann–Whitney Test*.

d *Fisher's exact test*.

*^e^The subset cohort is mainly used for association analysis and only have control group for the bacterial gene analysis (See Supplementary Methods)*.

### Metabolites in the Carnitine-TMAO Pathway in CVID and Healthy Controls

**Figure 2:**
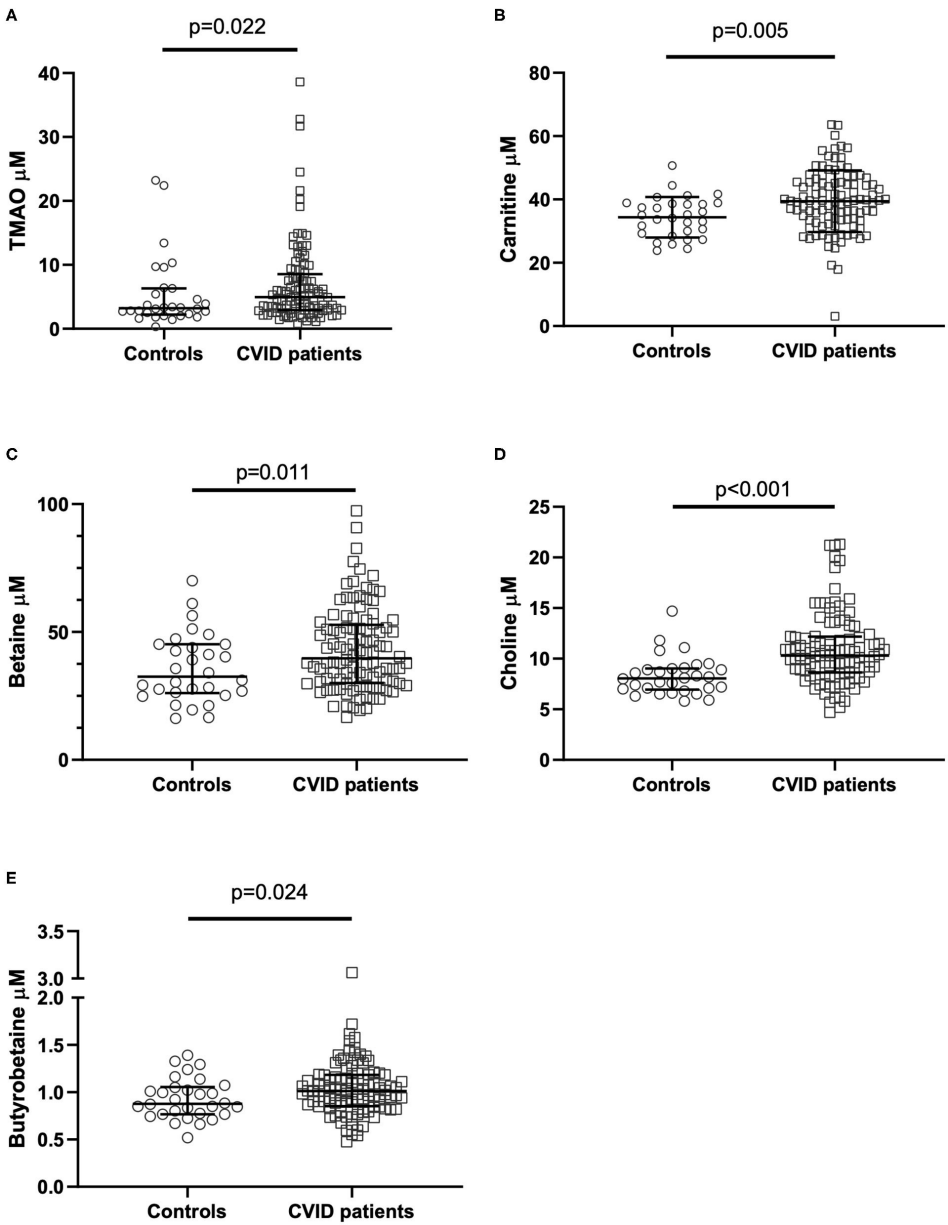
**(A–E)** TMAO and TMAO pathway metabolite levels in CVID patients and controls. Plasma levels of **(A)** TMAO, **(B)** carnitine, **(C)** betaine, **(D)** choline, **(E)** y-butyrobetaine in common variable immunodeficiency (CVID) patients (*n* = 104) and healthy controls (*n* = 30). *P*-values corrected for age and sex using stepwise linear regression analyses. Each dot represents one individual; bars represent median levels with interquartile range.

CVID patients (*n* = 104) had significantly elevated plasma concentrations of TMAO compared to healthy controls (Figure 2A). The TMA precursors carnitine, choline, and betaine, as well as the intermediate metabolite y-BB, were also significantly increased in CVID patients compared to healthy controls (Figures 2B–E). The seven outliers in the CVID group (with the highest plasma concentration of TMAO in Figure 2A) were tested with a gene panel for monogenic disease in relation to their primary immunodeficiency (24), but were all negative.

To study the temporal course and stability of carnitine-TMAO pathway metabolites, we measured the concentrations in 20 CVID patients (mean age 51.2 years ± 11.8 [SD], 13 [65%] females) three times over 8 weeks. Sixteen patients completed all three measurements. Applying the Friedman test, we discovered no significant changes in concentrations of TMAO (*p* = 0.444), carnitine (*p* = 0.144), betaine (*p* = 0.174), choline (*p* = 0.472), or y-BB (*p* = 0.646) during temporal testing (Supplementary Figure 1).

### TMAO in Relation to Systemic Inflammation

**Figure 3:**
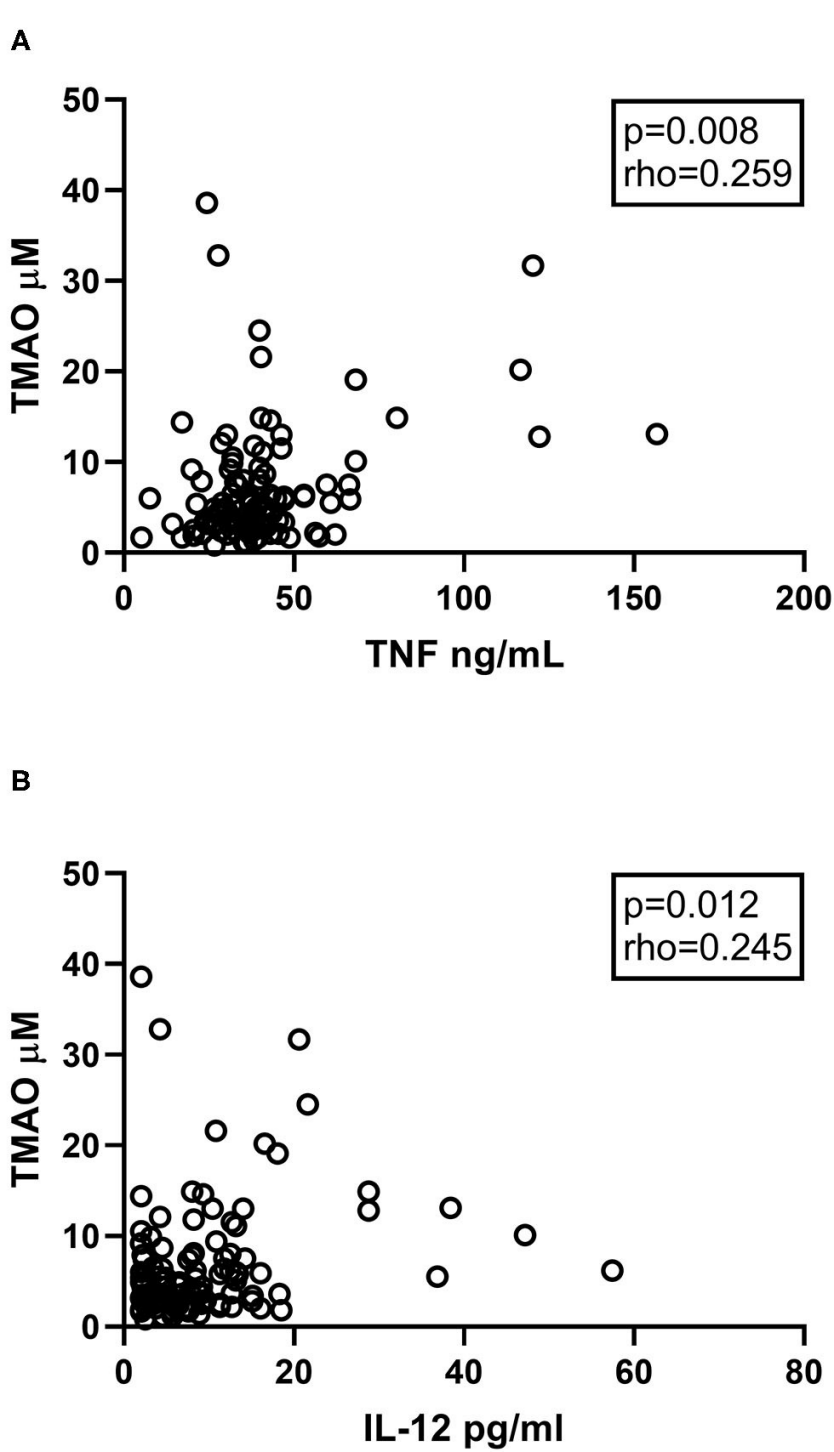
**(A,B)** Correlations between TMAO and inflammatory markers. Plasma levels of TMAO correlate positively with **(A)** TNFα and **(B)** IL-12 in CVID patients (*n* = 104). Two-tailed *p*-values were calculated using Spearman's rank correlation.

We and others have previously demonstrated increased inflammatory cytokines such as IL-6, IL-8, IL-12, and TNFα in CVID (25–27). These cytokines were all markedly elevated in the 104 CVID patients compared to controls (Supplementary Table 2) (8). We therefore selected these inflammatory markers to explore the association between systemic inflammation and TMAO. Plasma TMAO was positively associated with plasma levels of TNFα and IL-12 (Figures 3A,B), but not with IL-6 (*p* = 0.263, rho = 0.11) or IL-8 (*p* = 0.214, rho = 0.12). Data on other measured cytokines and their association to TMAO are shown in Supplementary Table 3.

### Metabolites of the Carnitine-TMAO Pathway in Relation to Gut Microbiota

**Figure 4:**
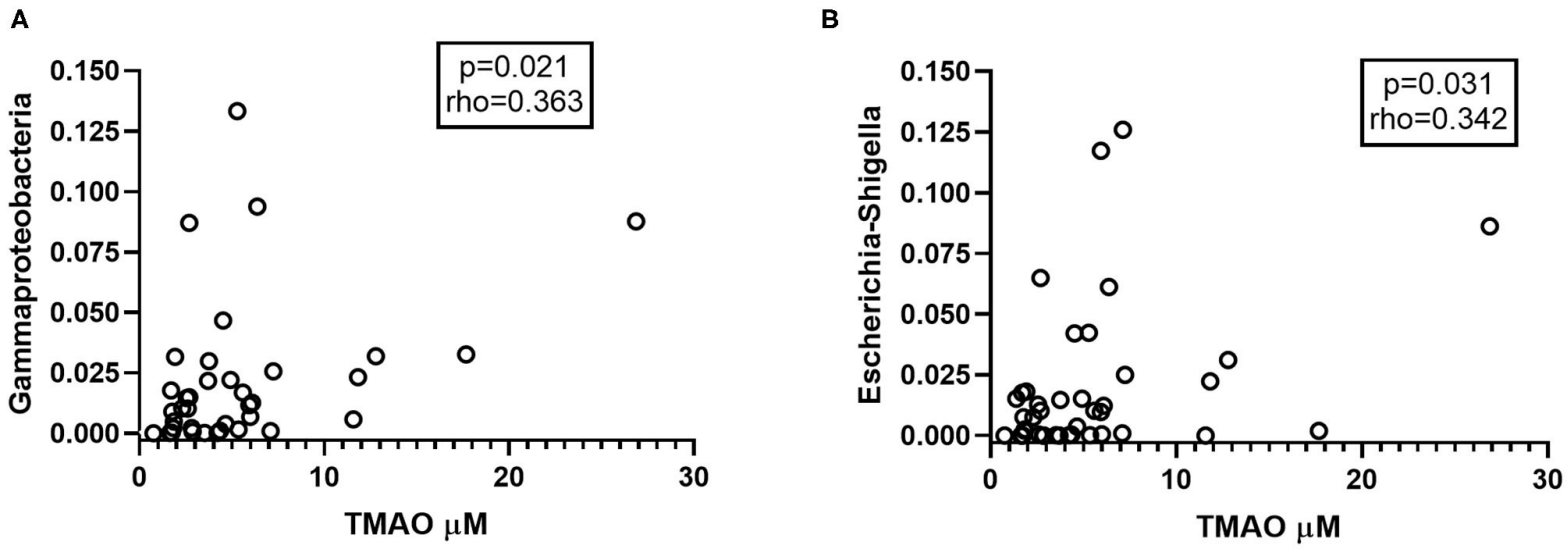
**(A,B)** Abundant taxa in CVID gut microbiota correlating with TMAO. **(A)** *Gammaproteobacteria* and **(B)** *Escherichia-Shigella*, taxa found in abundance in the gut microbiota of CVID patients, correlate positively with plasma levels of TMAO (*n* = 40). In panel **(A)** one outlier is not shown (*Gammaproteobacteria* 0.5, TMAO 1.4 μM). Two-tailed *p*-values were calculated using Spearman's rank correlation.

Using existing data on gut microbiota composition from a subset of the CVID patients (*n* = 40) (23), we explored if specific bacterial taxa previously found to differentiate between CVID patients and healthy controls correlated with TMAO pathway metabolites in plasma sampled at the same time point. These included the ten taxa making up the CVID dysbiosis index found to capture CVID dysbiosis in the gut: *Bacilli, Dorea, Roseburia, Gammaproteobacteria* (increased in CVID), and *Bifidobacterium, Odoribacteracea, Christensenellaceae, Blautia, Sutterella, Desulfovibrionacea* (reduced in CVID) (8). In addition, we included five taxa identified to differ between CVID and healthy controls in another publication using a different statistical approach (ANCOM): *Hungatella, Flavonifractor, Veillonella*, and *Escherichia-Shigella* (increased in CVID) and *Christensenellaceae R-7 group* (reduced in CVID) (28). The relative abundance of *Gammaproteobacteria* in stool samples from CVID patients correlated positively with concentrations of TMAO in plasma (Figure 4A). On genus level, we found positive correlations between abundance of *Escherichia-Shigella* in stool samples and plasma concentrations of TMAO (Figure 4B) and y-BB (*p* = 0.046, *r* = 0.32) in CVID patients. Both of these taxa were increased in CVID patients compared to controls in previous publications from our group (Supplementary Table 4). *Blautia*, a Gram positive bacterial genus previously shown to be reduced in CVID patients, was negatively correlated with the TMA precursor betaine (*p* = 0.037, *r* = −0.33).

### Bacterial Genes, CutC and CntA, in Stool Samples

**Figure 5:**
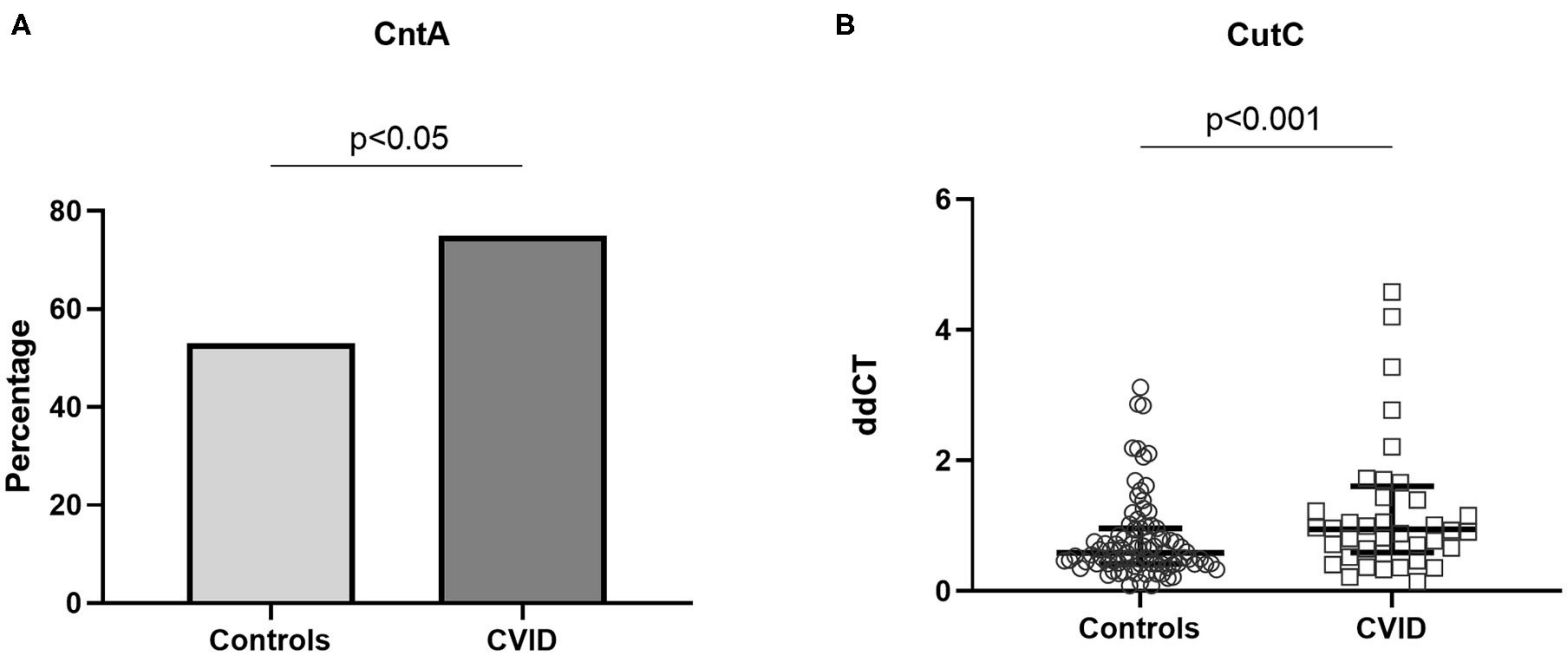
**(A,B)** Bacterial genes *CntA* and *CutC* in stool samples from CVID patients and controls. Using qPCR on DNA stool samples, *CntA* and *CutC* expression was explored in 40 CVID patients and 86 age-, sex, and BMI-matched controls. **(A)** *CntA* is expressed as a percentage of the number of individuals with *CntA* present in the two groups, Fisher's exact test. **(B)** *CutC* is presented as ddCT gene expression (*CutC* levels normalized to 16S level), results shown as median and IQR (Mann–Whitney). Three data points are outside the axis limits; one in the control group (ddCT 19.6) and two among the CVID patients (ddCT 10.8 and 30.5, respectively).

Bacterial genes that have been suggested to encode enzymes responsible for TMA production (29), *CutC* and *CntA*, were measured in stool samples from 40 CVID patients and 85 age-, sex, and body mass index (BMI)-matched controls (Supplementary Methods). These bacterial genes have also been reported to be associated with increased abundance of *Gammaproteobacteria*, demonstrated to be enriched in the gut microbiota from CVID patients (Supplementary Table 4). In a previous study, *CntA* was detected in only 26.0% of human samples whereas *CutC* was found in all human samples (12). Due to the anticipated low number of individuals that would have *CntA* present, we therefore compared the number of individuals that had *CntA* present in the two groups. Using qPCR on DNA stool samples, we found that *CntA* was present in significantly more CVID samples (*n* = 30, 75%), than controls (*n* = 45, 53%), *p* = 0.020 (Fisher's exact test, Figure 5A). As expected, *CutC* was present in all samples. For *CutC* we went on to measuring gene expression (ddCT) and found an increase in *CutC* gene expression in CVID patients compared to controls, *p* = 0.003 (Figure 5B). To conclude, both TMA producing bacterial genes; *CutC* and *CntA*, seem to be more prevalent in stool samples from CVID patients compared to controls.

We went on to exploring if there was any correlation between the expression of *CutC* in stool samples and the taxa making up the CVID dysbiosis index or the other the five taxa differing between CVID patients and controls (as defined above). We found that *CutC* levels were negatively correlated with *Bifidobacterium* (*p* = 0.04, rho = −0.33) and *Bacilli* (*p* = 0.028, rho = −0.35), but positively correlated with *Hungatella* (*p* = 0.003, rho = 0.46). *Bifidobacterium* is thought to have health-promoting properties and is used in probiotic supplements (30). We have previously found very low abundance of *Bifidobacterium* in CVID patients compared to controls (8), and the negative correlation with *CutC* could suggest that in CVID patients, TMA producing bacteria are increased at the expense of beneficial gut bacteria such as *Bifidobacterium*.

### TMAO in Relation to LPS and Serum IgA Levels

**Figure 6:**
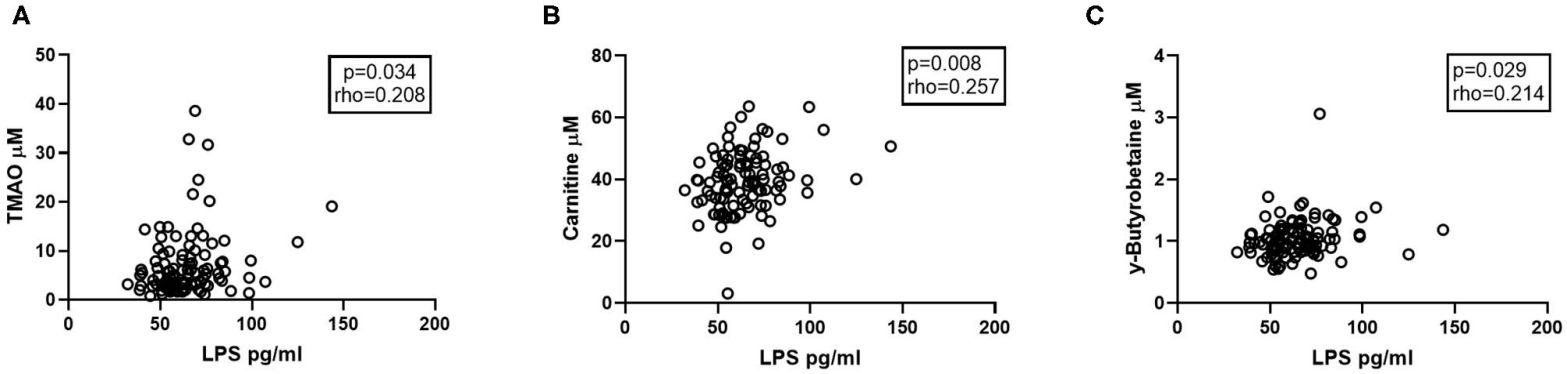
Correlations between carnitine-TMAO metabolites and LPS. Plasma levels of LPS correlate positively with **(A)** TMAO, **(B)** Carnitine, and **(C)** y-Butyrobetaine in CVID patients (*n* = 104). Two-tailed *p*-values were calculated using Spearman's rank correlation.

We have previously shown that there are increased amounts of the microbial product LPS in the plasma of patients with CVID (*n* = 104, Supplementary Table 2) (8), potentially contributing to systemic inflammation in these patients. Herein, we found that plasma levels of LPS were significantly correlated with TMAO and the TMA precursors carnitine and y-BB in CVID patients (Figures 6A–C), indicating a relation between gut leakage and TMAO pathway metabolites.

Similar to CVID, patients with selective IgA deficiency also have lower gut microbial diversity (28), thus it is of interest to explore whether undetectable serum IgA levels could influence TMAO concentrations in CVID patients. We compared CVID patients with undetectable serum IgA levels (IgA < 0.1 g/L) to CVID patients with detectable serum IgA levels (IgA ≥ 0.1g /L) and found no significant difference in plasma TMAO concentration between these two groups (*p* = 0.844, Supplementary Table 5). This may be due to a cut-off level for serum IgA of <0.1 g/L being too strict (28), and that low, but detectable, serum levels of IgA could still affect gut microbial composition in CVID, and thereby TMAO levels.

Furthermore, LPS and low serum IgA concentrations have been found to correlate with development of autoimmune cytopenias in CVID patients (31, 32). We found no significant difference in LPS levels between CVID patients with serum IgA < 0.1 g/L compared to CVID patients with IgA ≥ 0.1 g/L, or CVID patients with or without autoimmune cytopenia, organ specific autoimmunity or enteropathy (Supplementary Table 6). To further look into the potential role of LPS and undetectable serum IgA levels in the immunopathogenesis of CVID, we investigated certain inflammatory cytokines in relation to these markers. LPS correlated positively with IL-6 (*p* = 0.028, *r* = 0.216), a central cytokine in the pathogenesis of several autoimmune disorders as well as of inflammation in CVID (4), but not did not correlate significantly with IL-8 (*p* = 0.192, rho = 0.129), IL-12 (*p* = 0.064, rho = 0.182), or TNFα (*p* = 0.143, rho = 0.145). Additional correlation analyses between LPS and cytokines are given in Supplementary Table 7. Cytokine analyses of CVID patients with serum IgA < 0.1 g/L compared to CVID patients with IgA ≥ 0.1 g/L showed increased concentrations of two chemokines involved in inflammatory processes (i.e., IP-10 and MCP-1) in patients with IgA < 0.1 g/L, but did not display an overall different pattern in cytokine levels than those with IgA ≥ 0.1 g/L (Supplementary Table 8). To conclude, the link between LPS and inflammation in CVID could appear to occur on an overall group level. However, low IgA levels may be of greater importance than demonstrated here, conditional upon the cut-off used for low/undetectable levels of serum IgA.

### TMAO in Relation to Diet

A detailed self-reported food frequency questionnaire was obtained from 38 CVID patients. Food items that have been described to be associated with metabolites in the carnitine-TMAO pathway include meat, fish, eggs, and dairy products. CVID patients did not appear to consume more of these food items compared to a reference population (33) (Supplementary Figure 2). Hence, an increased consumption by CVID patients of egg, dairy products, meat, or fish did not appear to be responsible for the increased TMAO concentrations seen in these patients. Additionally, we investigated other food items relevant to gut microbiota and inflammation such as fiber, protein, wine, and sugar (34–36) for association analyses with TMAO. Wine consumption correlated negatively with TMAO (*p* = 0.023, rho = −0.37) in CVID patients, and when separating wine consumption into red wine and white wine, the negative correlation with TMAO, corrected for age and sex, remained significant only for red wine (*p* = 0.018, β = −0.332). We also found that intake of table sugar, but not total sugar consumption, was associated with increased plasma concentrations of TMAO (*p* = 0.006, rho = 0.44). In contrast, dietary intake of meat, fish, egg, dairy products, and fiber were not found significantly associated with TMAO in plasma (Supplementary Table 9), further supporting that other mechanisms than diet are responsible for the increased TMAO in CVID.

### TMAO in Relation to Rifaximin Use

It has previously been shown that a course of short-term antibiotics such as ciprofloxacin and metronidazole can reduce TMAO concentrations in plasma of healthy humans, as well as in mouse models of cardiovascular disease (administering vancomycin, neomycin sulfate, metronidazole, or ampicillin for 3 weeks) (37, 38). We have previously shown that a 2-week course of the broad spectrum oral antibiotic rifaximin alters gut microbial composition, but not systemic inflammation in CVID (23). Based on the knowledge that rifaximin acts purely locally in the gut and that several antibiotics have previously been demonstrated to influence TMAO concentrations, it was intriguing to investigate whether rifaximin (550 mg twice-daily) could have a similar impact. We therefore measured the effect of 2 week administration of rifaximin on plasma concentrations of TMAO in 40 CVID patients and found that there was no difference in TMAO concentrations between the rifaximin intervention group (*n* = 20) (baseline 5.0 [3.5–6.2] μmol/L, 2 weeks 4.3 [2.7–7.0] μmol/L, and 6 weeks 4.4 [3.1–6.9] μmol/L) and the no intervention group (*n* = 20) (baseline 3.2 [2.0–5.5] μmol/L, 2 weeks 5.5 [2.5–9.0] μmol/L, and 6 weeks 3.6 [1.9–11.2] μmol/L), *p* = 0.975 (UNIANOVA), data are given in median (25–75 percentile) (Supplementary Figure 3).

### TMAO in Relation to Clinical Characteristics of the CVID Patients

When dividing the CVID patients (*n* = 104) into two main subgroups by phenotype (i.e., infection only [*n* = 25] and those with non-infectious inflammatory and/or autoimmune complications [*n* = 79]), we found no differences in TMAO concentration between “infection only” (6.2 ± 4.0 μmol/L) vs. “non-infectious complications” (7.3 ± 7.3 μmol/L, mean ± SD), *p* = 0.922. Specific analyses of CVID subgroups including autoimmune cytopenia, organ-specific autoimmunity, and enteropathy did not show significant differences in TMAO concentration between patients with and without these features (Supplementary Table 9). Only two of the 104 CVID patients were not on Ig-replacement therapy and notably, these two were not outliers in the plasma TMAO concentration data (both had plasma TMAO concentration <15 μmol/L).

Both age and sex correlated significantly with metabolites in the carnitine-TMAO pathway in CVID patients and controls (*p* < 0.001, rho = 0.36 and *p* = 0.02, rho = 0.24, respectively), and were therefore adjusted for in statistical analyses (see Methods). BMI has previously been found to correlate positively with TMAO in some studies (39, 40), but we did not find this in our dataset (*p* = 0.802, rho = −0.03). Finally, kidney function could potentially influence TMAO concentrations. We therefore explored if there was any difference in estimated glomerular filtration rate (eGFR) between CVID patients and controls, which could potentially have been a confounding factor. We found no significant difference in eGFR between the patients and controls (59.4 ± 4.2 vs. 60.0 ± 0.0, mean ± SD), *p* = 0.277. Interestingly, within the CVID group, we found no significant difference in plasma TMAO concentration between those with eGFR < 60 vs. those with eGFR > 60 (11.1 ± 6.4 vs. 6.9 ± 6.7 μmol/L, mean ± SD, *p* = 0.082).

## Discussion

This is, to the best of our knowledge, the first study measuring TMAO and its related metabolites in CVID. The main findings of this study were: (i) CVID patients had persistently elevated plasma concentrations of TMAO and its precursors carnitine, choline, betaine as well as its intermediate metabolite y-BB compared to healthy controls; (ii) TMAO concentrations were positively correlated with TNFα and IL-12, representing prototypical inflammatory markers in CVID; (iii) The abundance of *Gammaproteobacteria* in stool and LPS in plasma were associated with increased concentrations of TMAO in CVID, furthermore bacterial genes *CntA* and *CutC* encoding TMA producing enzymes were more abundant in the gut microbiota of CVID patients than in controls. Our findings suggest that TMAO could be a link between disturbed gut microbiota and systemic inflammation in CVID.

The plasma concentrations of TMAO are influenced by several factors, including diet, gut microbiota, and kidney function (11, 41). In the present study, CVID patients were characterized by high TMAO concentrations, correlating with systemic inflammation and certain key bacteria related to the gut dysbiosis in CVID, as well as with LPS thought to reflect gut leakage mechanisms. In contrast, we did not find significant associations with kidney function or dietary intake of major food groups. Increased TMAO concentrations have previously been reported in several metabolic disorders, e.g., T2DM, obesity, and cardiovascular disease, and has also been shown to predict cardiovascular disease risk and outcome (42). However, increased TMAO concentrations have also been linked to other diseases such as HIV, irrespective of cardiovascular disease, where the increase in TMAO has been associated with altered gut microbial composition and systemic inflammation (43). Herein, we report a similar pattern in CVID, with increased TMAO concentrations associated with immune activation, gut microbial dysbiosis, and elevated LPS (Figure 1B).

A key question is whether TMAO is not only a marker but also a mediator of inflammation (44). Mechanistic studies have demonstrated that TMAO triggers activation of the NLRP3 inflammasome in endothelial cells, involving increased production of reactive oxygen species (45, 46). In addition, mice studies have demonstrated that dietary supplements of TMAO result in reduced reverse cholesterol transport *in vivo* (47), which could potentially promote inflammation. In line with this, we have recently shown that CVID patients with non-infectious complications have impaired reverse cholesterol transport, potentially contributing to systemic inflammation in these patients (48).

We found a positive association between abundance of *Gammaproteobacteria* and *Escherichia-Shigella* in the gut microbiome of CVID patients and concentrations of TMAO in plasma. Supporting our findings, *Gammaproteobacteria* have previously been shown to produce TMA in the gut of HIV-infected patients (43). Administration of oral rifaximin for 2 weeks did not significantly alter plasma TMAO concentrations in CVID patients, likely due to a lack of effect on central gut microbes (23) involved in TMA production. The increased amount of TMA producing enzyme *CntA* in stool samples from CVID patients compared to controls fits well with the association of increased *Gammaproteobacteria* and TMAO concentrations in CVID. However, since *CntA* amplicons display high identities (~99%) to *Gammaproteobacteria*-derived references (primarily from *Escherichia coli*) (12), it is difficult to separate the association with *CntA* from the association with *Gammaproteobacteria*. *CutC* amplicons have been associated with various taxa (e.g., *Clostridium XIVa* strains), but many sequences are not identified, suggesting that key human TMA producers are yet to be isolated (12). It is however important to remember that the abundance of *CntA* and *CutC* has previously been described to encompass <1% of the total bacterial community (12). The additional mechanism, where gut microbiota converts carnitine to TMA via the intermediate γBB, may be an equally important route for TMA formation from carnitine in the human gut (49). This is illustrated by the increased plasma concentrations of γBB in CVID compared to controls and the positive association of plasma γBB to *Escherichia-Shigella* in stool samples. Of note, bacterial genes involved in the two-step process with γBB are unknown and were therefore not investigated further (49).

Differences in intake of food items such as meat, fish, eggs, and dairy products, known to increase TMAO and its related metabolites cannot explain the increased TMAO concentration seen in CVID patients compared to controls. In the present study, there were no associations between the main components of the diet (i.e., meat, fish, dairy products, and fiber) and TMAO in the CVID patients. The negative correlation between consumption of red wine and plasma TMAO is however noteworthy, since red wine may contain compounds inhibiting of microbial TMA formation, such as DMB (3,3-dimethyl-1-butanol), reducing the potential for hepatic TMAO synthesis (50, 51). However, the lack of association of food items known to increase TMAO and related metabolites underscores that the high TMAO concentrations in CVID are not related to diet, but more likely related to gut microbial composition.

The strengths of this study, compared to other TMAO studies, are the inclusion of gut microbiota and dietary data allowing us to investigate carnitine-TMAO pathway metabolites in more detail. In addition, the total number of patients in the TMAO metabolite and inflammatory marker analyses was substantial, considering the prevalence of CVID. The limitations of this study are not having dietary information and gut microbial data on all CVID patients, as well as a small sized control group for the plasma/serum analyses compared to the control group for the microbiota analyses in stool. Furthermore, associations do not necessarily signify causal relationship, thus mechanistic studies are needed to further explore the role of TMAO in persistent inflammation and immune activation such as seen in CVID.

In conclusion, plasma TMAO concentrations are elevated in CVID patients compared to healthy controls, correlating with markers of systemic inflammation and gut microbial abundance of *Gammaproteobacteria*. These patients also have increased amounts of gut bacterial genes *CntA* and *CutC*, encoding TMA producing enzymes. We suggest that TMAO could be a link between disturbed gut microbiota and systemic inflammation in CVID, and that its relation to certain key bacteria indicates that gut microbiota could be a therapeutic target to reduce TMAO formation and thereby systemic inflammation in CVID.

## Data Availability Statement

The datasets analyzed during the current study are not publicly available due to Norwegian legislation regarding general data protection regulation, but are available from the corresponding author on reasonable request.

## Ethics Statement

The studies involving human participants were reviewed and approved by The Regional Committee for Medical and Research Ethics, South East Norway. The patients/participants provided their written informed consent to participate in this study.

## Author Contributions

MM, PA, BF, and SJ designed research. KH, RB, TM, and TD conducted research. JH, MK, KO, and BH provided essential materials. MM, TU, and SJ performed statistical analysis. MM, JH, MK, MT, PA, BF, and SJ analyzed data. MM and SJ wrote the paper and had primary responsibility for the final content. All authors revised the manuscript for critical content and approved the final version.

## Conflict of Interest

JH has served on advisory boards for Orkla Health and Novartis, and received research support from Biogen, all unrelated to the present study. The remaining authors declare that the research was conducted in the absence of any commercial or financial relationships that could be construed as a potential conflict of interest.

## Abbreviations

BMI: body mass index
*CntA*: carnitine oxygenase
*CutC*: choline-TMA lyase
CVID: Common variable immunodeficiency
FFQ: food frequency questionnaire
Ig: immunoglobulins
IL: interleukin
LPS: lipopolysaccharide
TMA: trimethylamine
TMAO: trimethylamine N-oxide
TML: trimethyllysine
TNF: tumor necrosis factor
γBB: γ-butyrobetaine.
