# Supplementary material for: Gut Microbiota-Dependent Trimethylamine N-Oxide Associates With Inflammation in Common Variable Immunodeficiency
Source: Front Immunol. 2020 Sep 16;11:574500. doi: 10.3389/fimmu.2020.574500 (PMC7525000; doi:10.3389/fimmu.2020.574500)
Supplement: Supplementary file 1 [file Data_Sheet_1.PDF]

Supplementary Material

Table of content:

Supplementary Methods

Supplementary Figure S1

Supplementary Figure S2

Supplementary Figure S3

Supplementary Table S1

Supplementary Table S2

Supplementary Table S3

Supplementary Table S4

Supplementary Table S5

Supplementary Table S6

Supplementary Table S7

Supplementary Table S8

Supplementary Table S9

References

Supplementary Methods

Gut microbiota analyses

The sequencing was performed on an Illumina MiSeq. Briefly, the hypervariable V3-V4 region was amplified with generic primers as described in detail(1), including the gene specific primer sequences CCTACGGGAGGCAGCAG (forward) and GGACTACHVGGGTWTCTAAT (reverse) and up to 192 (24x8 barcodes) samples in parallel. Cleanup and normalization were performed using the SequalPrep Normalization Plate Kit (Life Technologies), followed by pooling and quality control. The final libraries were sequenced on an Illumina MiSeq (Norwegian Sequencing Centre, Oslo University Hospital Ullevål) using the v3 kit, allowing up to 300 basepairs paired-end sequencing. Paired-end reads were quality trimmed with cutadapt version 1.13 and then merged using FLASH version 1.2.11. The merged reads were de-multiplexed and quality filtered using default values in Quantitative Insights Into Microbial Ecology (QIIME) version 1.9.1. Closed reference operational taxonomic unit (OTU) mapping to the Silva database (version 123, reference OTUs clustered at 97% sequence similarity) was performed using SortMeRNA version 2.0 through QIIME. A rarefied OTU table (9525 reads per sample) was generated and OTUs with less than 2 reads to support it were discarded.

Measurement of carnitine, TMAO and related metabolites

Free carnitine and γBB, were analyzed in plasma using MS/MS as described previously (2) with some modifications of the high-performance liquid chromatography (HPLC) conditions: The LC system was an Agilent (Waldbronn, Germany) 1200 Series with binary pump, variable volume injector, and a thermostated autosampler. HPLC separation was conducted at 30 °C using a gradient solvent mixture. Mobile phase A was made of 10 mM ammonium acetate and 12 mM heptafluorobutyric acid (HFBA) in water, and mobile phase B was made of 10 mM ammonium acetate and 12 mM HFBA in methanol. The gradient was B 0.1 min 20%, flow 0.2 mL/min; B 4 min 20–90%, flow 0.2 mL/min; B 14 min 90%, flow 0.2 mL/min; B 10 min 2%, flow 0.6 mL/min; B 0.1 min 20%, flow 0.2 mL/min. A Phenomenex Luna C8 column (5 μm, 150 × 2 mm) equipped with a Phenomenex C18 pre-column, (4.0 × 2.0 mm) was used. Two μl of the sample were injected. Levels of TMAO, choline and betaine were measured using the same assay as used to determine the concentrations of carnitine. Stable isotope dilution liquid chromatography–tandem mass spectrometry (LC/MS/MS) was used for the quantification of TMAO, choline and betaine; all three were monitored in positive liquid chromatography–tandem mass spectrometry (MRM) MS mode using characteristic precursor–product ion transitions: m/z 76→58, m/z 104→60 and m/z 118→58, respectively. The internal standards TMAO-trimethyl-d9 (d9-TMAO), choline-trimethyl-d9 (d9-choline) and betaine-trimethyl-d9-methylene-d2 (d11-betaine) were added to plasma samples before protein precipitation and were similarly monitored in MRM mode at m/z 85→66, m/z 113→69 and m/z 129→66, respectively. Various concentrations of TMAO, choline and betaine standards and a fixed amount of internal standards were spiked into 4% bovine serum albumin to prepare the calibration curves for the quantification of plasma analytes. All stable isotope-labelled internal standards were purchased from Cambridge Isotope Laboratories, Inc. (Andover, MA).

Patient's and control's characteristics for the gut microbiota, *CutC* and *CntA* analyses

The CVID samples for the *CutC* and *CntA* analysis were baseline samples (n=40) from a previous published intervention study with rifaximin (3), and the same baseline samples were used for the taxonomic gut microbiota analysis in this study. Since the intervention study did not include a control population, the control group for these analyses was selected from a control cohort that had previously been published in two separate studies in 2016 as a control

group for primary sclerosing cholangitis and CVID, respectively (4, 5). The number of controls was aimed at approximately 2:1, and in the end 86 age-, sex and BMI-matched controls were selected. Only age, sex and BMI were used for selection criteria for controls, and the selection was done before the *CutC* and *CntA* analysis was performed. There was no statistical difference in age (49 [±12] vs 50 [±5], p= 0.78), BMI (26 [±5] vs 26 [±4], p=0.13); given in median and standard deviation) or sex (37 % men vs 38 % men, p=1.00) between CVID patients and controls, respectively. The controls were randomly selected from donors registered in the national Norwegian Bone Marrow Donor Registry (Oslo, Norway) and were contacted by a letter and asked to send a stool sample which was collected by the participants at home and sent by air mail. Therefore, this control-group does not have any corresponding blood samples.

Detailed information on the rifaximin study

Forty CVID patients (age 21-69 years old) were included to a randomized, open, prospective, single-center, clinical trial (the rifaximin study) at Oslo University Hospital, Rikshospitalet, Norway, between Oct 8 2013, and Oct 20, 2014. For these CVID patients, exclusion criteria were: antibiotics in the last 12 weeks, history of allergic reaction to rifaximin, malignancy, impaired kidney function, pregnancy or lactation, on-going infection, use of probiotics in the last 6 months, immunosuppressive drugs, comorbidity that may influence with the patient's safety or compromise the study results (e.g., cardiovascular disorders, alcoholism, psychiatric disease, HIV infection), and polypharmacy (patient with an extensive medication list i.e. ten drugs or more). The CVID patients were randomized by computer-generated randomization (1:1) into two groups (*rifaximin n=20* and *no intervention n=20*) and there was no placebo drug (3).

Supplementary Figures

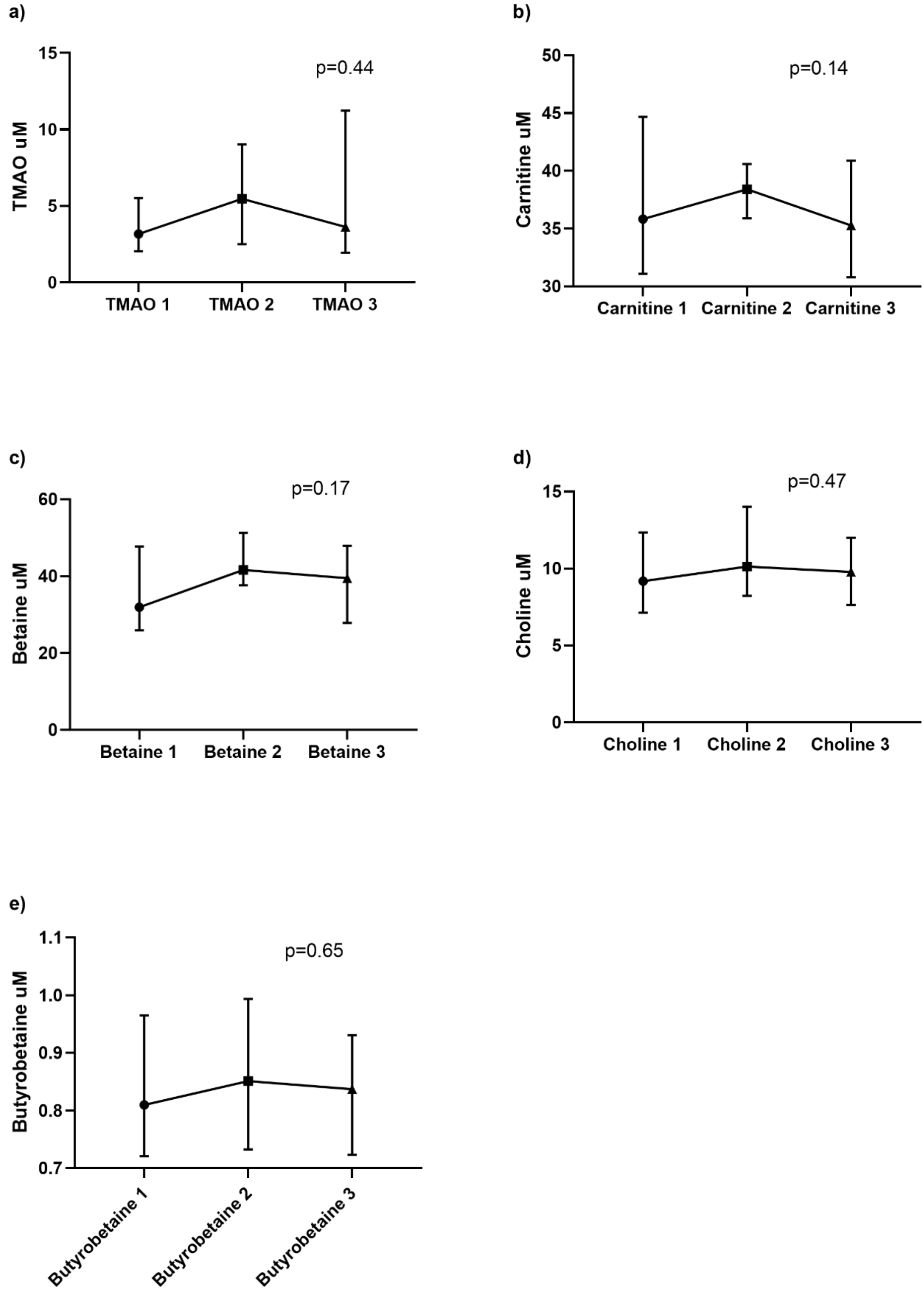

Supplementary Figure S1: Temporal testing of TMAO pathway metabolites

Plasma levels over three time points (0, 2 and 8 weeks) for (A) TMAO, (B) Carnitine, (C) Betaine, (D) Choline, (E) ɣ-butyrobetaine in CVID patients (n=16). P-values calculated using the Friedman test. Results are shown as median with interquartile range.

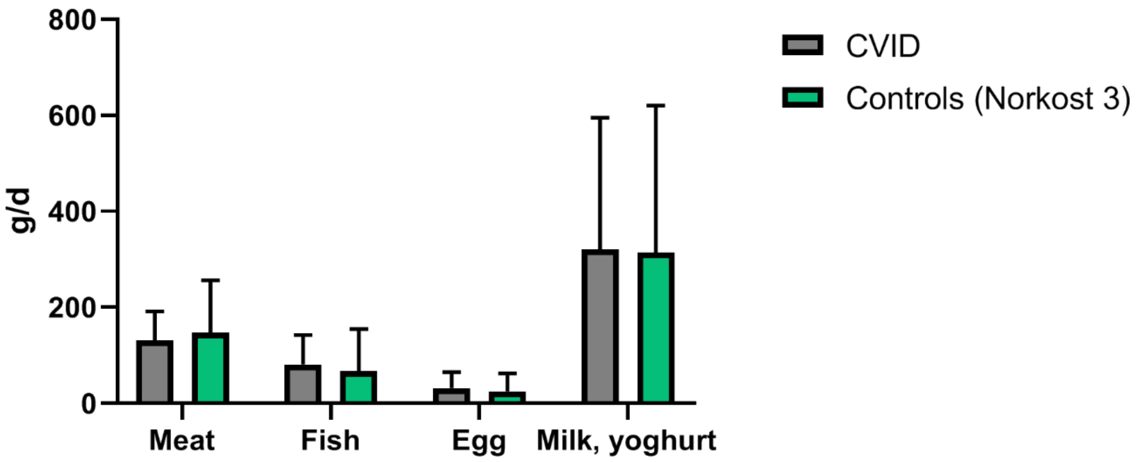

Supplementary Figure S2: Dietary intake amongst CVID patients vs. background population

Daily intake of meat, fish, egg, milk and yoghurt in the CVID patient cohort (n=38) versus the Norwegian background population (Norkost3, n=1787). Results shown as mean with SD. We found non-significant differences between the groups when applying multiple t-test analysis for meat (p=0.37), fish (p=0.33), egg (p=0.27) and milk/yoghurt (p=0.89) intake. The Norkost3 study used a 24 hour recall questionnaire, aiding accuracy of reported diet, whilst using a high *n* to reduce the seasonal and day-to-day variation.

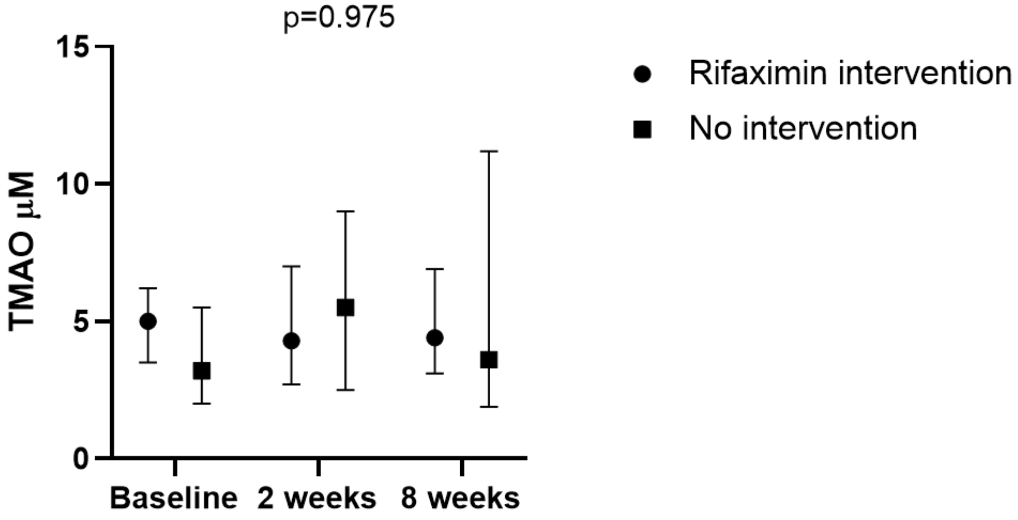

Supplementary Figure S3: The effect of a 2-week course of the oral antibiotic rifaximin on TMAO concentration

The figure shows plasma TMAO concentrations at baseline, 2 weeks and after an additional 6-week follow up period for CVID patients given 550 mg rifaximin twice-daily for two weeks from baseline (n=20) compared to CVID patients that were not given this intervention (n=20). Results given in median (25–75 percentile), p-value calculated using UNIANOVA.

Supplementary Tables

Supplementary Table S1: Autoimmune and inflammatory complications in the large and small CVID cohort

	**Complications to CVID in Main cohort** (n=104)	**Complications to CVID in Subset cohort** (n=40)
Infection only	25 (24)[a]	8 (20)
Splenomegaly	53 (51)	16 (40)
Enteropathy	27 (26)	13 (33)
Granulomas	17 (16)	6 (15)
Organ specific autoimmunity	20 (19)	8 (20)
Autoimmune cytopenia	22 (21)	8 (20)
Lymphoid hyperplasia	51 (49)	23 (56)
Nodular regenerative hyperplasia, liver	4 (4)	1 (2.5)
Lymphocytic interstitial pneumonitis	2 (2)	1 (2.5)

CVID patients divided into subgroups of infection only and various non-infectious complications as previously defined by H. Chapel et al(6). [a]Data given as n (%).

Supplementary Table S2: Inflammatory markers and LPS in CVID and controls

Independent variable	Median CVID	Range min-max CVID	Median controls	Range min-max controls	P-value
TNFα (pg/ml)	37.2	5.1-156.7	8.9	2.4-43.6	<0.001
IL-12 (pg/ml)	7.5	2.0-57.5	2.0	2.0-219.4	<0.001
IL-8 (pg/ml)	8.2	4.3-35.4	6.0	2.0-11.6	<0.001
IL-6 (pg/ml)	5.0	1.0-42.9	1.9	0.8-8.2	<0.001
LPS (pg/ml)	62.1	32.2-143.6	53.7	43.9-73.9	0.004

P-values given for linear regression using log-transformed independent variables correcting for age and sex (which were non-significant variables in this model) for inflammatory markers (data previously published, doi: 10.1038/mi.2016.18)(5). P-value given for Student's t-test using log-transformed variable for LPS. Median and range given in absolute values for each independent variable.

Supplementary Table S3: TMAO correlations with cytokines in CVID patients

Cytokine	Corr. coefficient	P-value
IL-1b	0.174	0.078[b]
IL-1ra	0.235	**0.016**[b]
IL-4	0.169	0.086[a]
IL-5	0.126	0.202[a]
IL-7	0.099	0.319[b]
IL-9	0.189	0.054[b]
IL-10	0.278	**0.004**[a]
IL-13	0.106	0.285[a]
IL-17a	0.165	0.094[b]
G-CSF	0.185	0.060[a]
IFNγ	0.159	0.106[a]
IP-10	-0.007	0.942[b]
MCP-1	0.068	0.495[b]
MIP-1a	0.213	**0.030**[a]
MIP-1b	-0.096	0.334[a]
RANTES	0.025	0.801[b]
VEGF	0.252	**0.010**[b]

Correlation analyses to log-transformed TMAO by Pearson's or Spearman's correlation applied as appropriate. [a]Pearson's correlation. [b]Spearman's correlation. $P<0.05$ in bold.

Supplementary Table S4: Gammaproteobacteria abundance in CVID patients and controls across three separate statistical approaches

Taxon	Increased in:	LDA Effect size	LEfSe *P*-value	MannW Q-value	MaAsLin Q-value
Bacteria.Proteobacteria.Gammaproteobacteria	CVID	3.4	$2.9x10^{-7}$	$1.2x10^{-5}$	0.0240
Bacteria.Proteobacteria.Gammaproteobacteria.Enterobacteriales	CVID	3.3	$1.6x10^{-6}$	$4.6x10\text{-}^{-5}$	0.0330
Bacteria.Proteobacteria.Gammaproteobacteria.Enterobacteriales.Enterobacteriaceae	CVID	3.3	$1.6x10^{-6}$	$4.6x10\text{-}^{-5}$	0.0330

LEfSe; The linear discriminant analysis (LDA) effect size, MannW; Mann Whitney U test, MaAsLin; Multivariate Association with Linear Model. These data have previously been published (doi: 10.1038/mi.2016.18)(5).

Supplementary Table S5: LPS in CVID subgroups

	LPS (pg/mL)		
**CVID subgroup**	**Yes**	**No**	**P-value**
IgA <0.1 g/L	65.9 [61.0, 70.8], (n=63)	60.7 [55.8, 65.6], (n=30)	0.232[a]
Autoimmune cytopenia	63.1 [59.0, 67.2], (n=22)	64.5 [60.4, 68.7], (n=82)	0.931[a]
Organspecific autoimmunity	60.8 [55.3, 66.2], (n=20)	65.1 [61.1, 69.0], (n=84)	0.399[a]
Enteropathy	65.2 [60.0, 70.4], (n=27)	63.9 [59.7, 68.1], (n=77)	0.515[a]

Results given as mean [95% CI] with *n* in brackets. IgA data are missing for 11 CVID patients. [a]Student's t-test.

Supplementary Table S6: LPS correlations with cytokines in CVID patients

Cytokine	Corr. coefficient	P-value
IL-1b	0.091	0.356[b]
IL-1ra	0.184	0.061[b]
IL-4	0.159	0.108[a]
IL-5	0.119	0.227[a]
IL-7	-0.017	0.866[b]
IL-9	0.275	**0.005**[b]
IL-10	0.129	0.192[a]
IL-13	0.281	**0.004**[a]
IL-17a	0.165	0.095[b]
G-CSF	0.169	0.086[a]
IFNγ	0.168	0.089[a]
IP-10	0.006	0.954[b]
MCP-1	0.037	0.707[b]
MIP-1a	0.156	0.114[a]
MIP-1b	0.209	**0.033**[a]
RANTES	-0.144	0.145[b]
VEGF	0.158	0.108[b]

Correlation analyses between cytokines and LPS using Pearson's or Spearman's correlation as appropriate. [a]Pearson's correlation. [b]Spearman's correlation. $p<0.05$ in bold.

Supplementary Table S7: Cytokines in CVID patients with serum IgA ≥0.1 g/L and serum IgA <0.1 g/L

Cytokine	IgA ≥0.1 g/L (n=30)	IgA <0.1 g/L (n=63)	P-value
IL-1b	1.8 ± 0.7	1.8 ± 0.9	0.513[b]
IL-1ra	92.8 ± 53.5	90.6 ± 64.3	0.310[b]
IL-4	2.7 ± 1.4	2.4 ± 1.1	0.345[a]
IL-5	3.2 ± 1.2	3.4 ± 1.5	0.622[a]
IL-6	5.8 ± 3.8	6.8 ± 6.4	0.628[b]
IL-7	5.0 ± 2.0	5.0 ± 2.9	0.423[b]
IL-8	9.6 ± 4.7	10.6 ± 6.1	0.565[b]
IL-9	28.3 ± 29.4	26.3 ± 32.6	0.400[b]
IL-10	7.0 ± 3.7	7.8 ± 6.4	0.998[a]
IL-12p70	10.4 ± 8.2	9.4 ± 10.3	0.248[b]
IL-13	10.3 ± 6.4	12.4 ± 16.5	0.943[a]
IL-17a	77.3 ± 48.9	68.9 ± 62.1	0.292[a]
G-CSF	60.9 ± 22.5	64.7 ± 34.8	0.980[b]
IFNɣ	97.6 ± 54.2	92.9 ± 74.0	0.568[a]
IP-10	784.9 ± 679.9	1120.3 ± 939.8	**0.014**[b]
MCP-1	3.8 ± 2.8	4.7 ± 2.8	**0.019**[b]
MIP-1a	3.4 ± 1.5	3.3 ± 2.0	0.812[a]
MIP-1b	18.9 ± 9.5	21.5 ± 13.7	0.544[a]
RANTES	6191.3 ± 2030.0	5814.3 ± 1982.2	0.316[b]
TNF	40.5 ± 19.2	41.5 ± 24.4	0.669[b]
VEGF	13.1 ± 10.5	11.0 ± 11.7	0.155[b]

Results are given as mean ±SD. [a]Student's t-test. [b]Mann-Whitney. p<0.05 in bold.

Supplementary Table S8: Correlations between TMAO and dietary intake

**Food item**	**TMAO** *P-value*	*Spearman's rho*
Meat	0.68	-0,07
Fish	0.72	0.06
Egg	0.14	0.25
Dairy products	0.28	-0.18
Fiber	0.77	-0.05

Supplementary Table S9: TMAO in subgroups of CVID patients

	**TMAO** (µmol/L **)**		
**CVID subgroup**	**Yes**	**No**	**P-value**
IgA <0.1 g/L	7.3 [5.4, 9.1], (n=63)	7.1 [4.9, 9.4], (n=30)	0.844[a]
Autoimmune cytopenia	7.5 [2.9, 12.1], (n=22)	6.9 [5.7, 8.1], (n=82)	0.068[b]
Organspecific autoimmunity	6.8 [4.4, 9.1], (n=20)	7.1 [5.5, 8.6], (n=84)	0.831[a]
Enteropathy	7.6 [4.7, 10.5], (n=27)	6.8 [5.3, 8.3], (n=77)	0.804[a]

Results given as mean [95% CI] with *n* in brackets. IgA data missing for 11 CVID patients. [a]Student's t-test. [b]Mann-Whitney.

References

1. Kozich JJ, Westcott SL, Baxter NT, Highlander SK, Schloss PD. Development of a dual-index sequencing strategy and curation pipeline for analyzing amplicon sequence data on the MiSeq Illumina sequencing platform. Appl Environ Microbiol. 2013;79(17):5112-20.
2. Vigerust NF, Bohov P, Bjorndal B, Seifert R, Nygard O, Svardal A, et al. Free carnitine and acylcarnitines in obese patients with polycystic ovary syndrome and effects of pioglitazone treatment. Fertil Steril. 2012;98(6):1620-6.e1.
3. Jørgensen SF, Macpherson ME, Bjornetro T, Holm K, Kummen M, Rashidi A, et al. Rifaximin alters gut microbiota profile, but does not affect systemic inflammation - a randomized controlled trial in common variable immunodeficiency. Sci Rep. 2019;9(1):167.
4. Kummen M, Holm K, Anmarkrud JA, Nygard S, Vesterhus M, Hoivik ML, et al. The gut microbial profile in patients with primary sclerosing cholangitis is distinct from patients with ulcerative colitis without biliary disease and healthy controls. Gut. 2017;66(4):611-9.
5. Jørgensen SF, Troseid M, Kummen M, Anmarkrud JA, Michelsen AE, Osnes LT, et al. Altered gut microbiota profile in common variable immunodeficiency associates with levels of lipopolysaccharide and markers of systemic immune activation. Mucosal Immunol. 2016;9(6):1455-65.
6. Chapel H, Lucas M, Lee M, Bjorkander J, Webster D, Grimbacher B, et al. Common variable immunodeficiency disorders: division into distinct clinical phenotypes. Blood. 2008;112(2):277-86.
